# (*E*)-Methyl 3-(4-ethyl­phen­yl)-2-{2-[(*E*)-(hy­droxy­imino)­meth­yl]phen­oxy­meth­yl}acrylate

**DOI:** 10.1107/S1600536811038359

**Published:** 2011-09-30

**Authors:** E. Govindan, K. SakthiMurugesan, J. Srinivasan, M. Bakthadoss, A. SubbiahPandi

**Affiliations:** a Department of Physics, Presidency College (Autonomous), Chennai 600 005, India; b Department of Organic Chemistry, University of Madras, Guindy Campus, Chennai 600 025, India

## Abstract

In the title compound, C_20_H_21_NO_4_, the two benzene rings are almost perpendicular to each other, making a dihedral angle of 86.1 (7)°. The hy­droxy­ethanimine group is essentially coplanar with the benzene ring, the largest deviation from the mean plane of the hy­droxy­ethanimine [C=N—OH] group being 0.011 (1) Å for the O atom. An intra­molecular C—H⋯O hydrogen bond occurs. The mol­ecules are linked into cyclic centrosymmetric *R*_2_^2^(6) dimers *via* O—H⋯N hydrogen bonds. Inter­molecular C—H⋯O hydrogen bonds link the mol­ecules, forming a *C*(8) chain along the *a* axis. The crystal packing is further stabilized by C—H⋯π inter­actions.

## Related literature

For structures of other acrylate derivatives, see: Zhang *et al.* (2009 ▶); Wang *et al.* (2011 ▶); SakthiMurugesan *et al.* (2011 ▶). For the use of oxime ligands in coordination chemistry, see: Chaudhuri (2003 ▶).
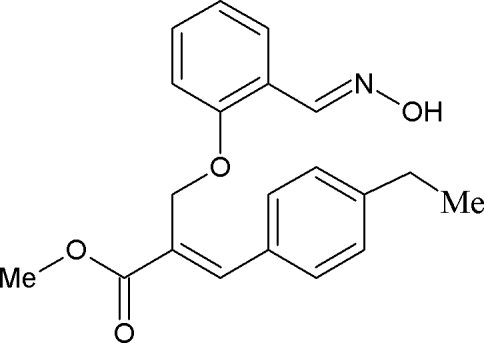


## Experimental

### 

#### Crystal data

C_20_H_21_NO_4_
*M* *_r_* = 339.38
Triclinic, 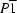

*a* = 9.0053 (2) Å
*b* = 9.3655 (3) Å
*c* = 12.1793 (3) Å
α = 75.299 (1)°
β = 74.756 (1)°
γ = 64.891 (1)°
*V* = 885.43 (4) Å^3^
*Z* = 2
Mo *K*α radiation
μ = 0.09 mm^−1^
*T* = 293 K
0.25 × 0.22 × 0.19 mm

#### Data collection

Bruker APEXII CCD area-detector diffractometer
Absorption correction: multi-scan (*SADABS*; Sheldrick, 1996 ▶) *T*_min_ = 0.978, *T*_max_ = 0.983
24628 measured reflections
6955 independent reflections
4453 reflections with *I* > 2σ(*I*)
*R*_int_ = 0.023

#### Refinement

*R*[*F* ^2^ > 2σ(*F* ^2^)] = 0.053
*wR*(*F* ^2^) = 0.189
*S* = 1.02
6955 reflections
229 parameters
H-atom parameters constrained
Δρ_max_ = 0.31 e Å^−3^
Δρ_min_ = −0.29 e Å^−3^

### 

Data collection: *APEX2* (Bruker, 2007 ▶); cell refinement: *SAINT* (Bruker, 2007 ▶); data reduction: *SAINT*; program(s) used to solve structure: *SHELXS97* (Sheldrick, 2008 ▶); program(s) used to refine structure: *SHELXL97* (Sheldrick, 2008 ▶); molecular graphics: *ORTEP-3* (Farrugia, 1997 ▶); software used to prepare material for publication: *SHELXL97* and *PLATON* (Spek, 2009 ▶).

## Supplementary Material

Crystal structure: contains datablock(s) global, I. DOI: 10.1107/S1600536811038359/nk2110sup1.cif

Structure factors: contains datablock(s) I. DOI: 10.1107/S1600536811038359/nk2110Isup2.hkl

Supplementary material file. DOI: 10.1107/S1600536811038359/nk2110Isup3.cml

Additional supplementary materials:  crystallographic information; 3D view; checkCIF report

## Figures and Tables

**Table 1:** Hydrogen-bond geometry (Å, °) *Cg*2 is the centroid of the C13–C18 ring.

*D*—H⋯*A*	*D*—H	H⋯*A*	*D*⋯*A*	*D*—H⋯*A*
O1—H1⋯N1^i^	0.82	2.15	2.8568 (15)	145
C14—H14⋯O2	0.93	2.51	3.3002 (16)	143
C15—H15⋯O4^ii^	0.93	2.50	3.3524 (16)	152
C5—H5⋯*Cg*2^iii^	0.93	2.94	3.7756 (14)	150

Symmetry codes: (i) 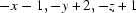; (ii) 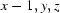; (iii) 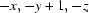.

**Fig. 1.**
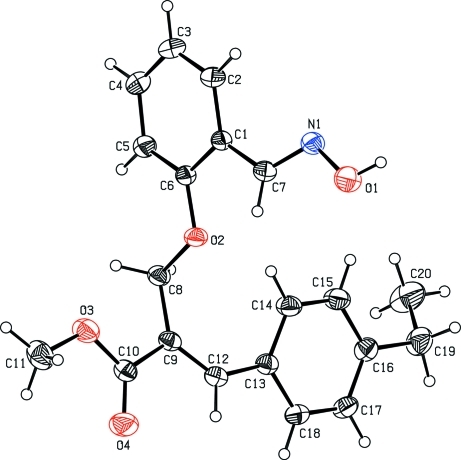
The title compound with displacement ellipsoids at the 30% probability level.

**Fig. 2.**
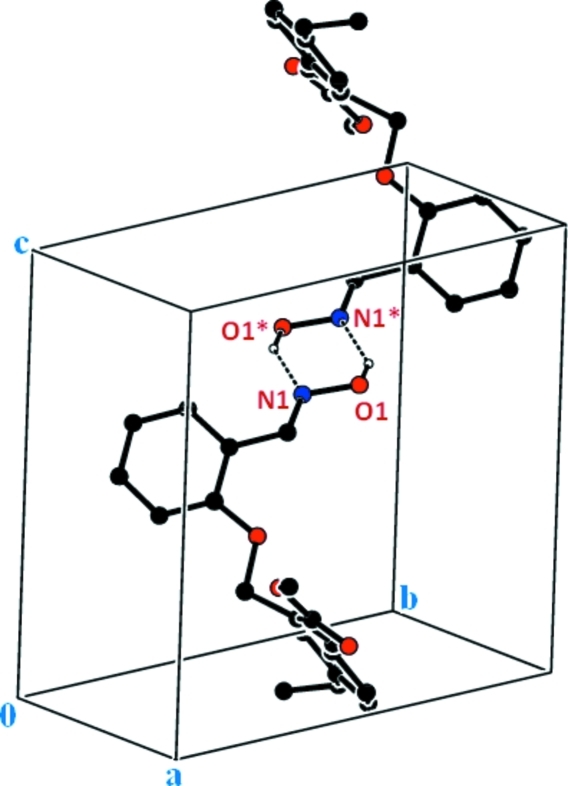
The crystal structure showing the centrosymmetric hydrogen bond motif R22(6). H atoms not involved in the motif have been omitted. Atoms marked with an asterisk (*) are at the symmetry position (-1 - x, 2 - y, 1 - z). Dashed lines indicate the hydrogen bonds.

## supplementary crystallographic information

### Comment

Recently, 2-cyanoacrylates have been extensively used as agrochemicals because of their unique mechanism of action and good environmental profiles (Zhang *et al.*, 2009). Oximes are a classical type of chelating ligands which are widely used in coordination and analytical chemistry (Chaudhuri, 2003). Against this background, and in order to obtain detailed information on molecular conformations in the solid state, an X-ray study of the title compound was carried out.

X-Ray analysis confirms the molecular structure and atom connectivity as illustrated in Fig. 1. The bond lengths and angles in (Fig. 1) agree with those observed in other acrylate derivatives (Wang *et al.*, 2011). The whole molecule is not planar as the dihedral angle between the two aryl rings is 86.1 (7)°, it shows that both the rings are almost perpendicular to each other. The oxime group having the C═N forming an E configuration. The hydroxyethanimine group is essentially coplanar with the benzene ring, the largest deviation from the mean plane of the hydroxyethanimine [C═N—OH] group is 0.011 (1)Å for the O1 atom.

The enoate group assumes an extended conformation as can be seen from torsion angles C9—C10—O3—C11 [178.1 (1) °] and C12—C9—C10—O3 [171.6 (1) °]. The atom C15 in the molecule (*x*,*y*,*z*) donate one proton to atom O4 of the molecule at (-1 + *x*,*y*,*z*) forming a C(8) chain along *a* axis. The hydroxyethanimine group in the molecules are linked into cyclic centrosymmetric dimers *via* O—H···N hydrogen bonds with the motif *R*_2_^2^(6) (Figure 2). In addition to van der Waals interaction, the crystal packing is stabilized by C–H..O, O–H···N and C–H···π interactions.

### Experimental

To a stirred solution of (*E*)-methyl 2-((2-formylphenoxy)methyl) -3-(4-ethylphenyl)acrylate (4 mmol) in 10 ml of EtOH/H_2_O mixture (1:1) was added NH_2_OH.HCl (6 mmol) in the presence of 50% NaOH at room temperature. Then the reaction mixture was allowed to stir at room temperature for 1.5 h. After completion of the reaction, solvent was removed and the crude mass was diluted with water (15 ml) and extracted with ethyl acetate (3 *x* 15 ml). The combined organic layer was washed with brine (2 *x* 10 ml) and dried over anhydrous Na_2_SO_4_ and then evaporated under reduced pressure to obtain (*E*)-methyl3- (4-ethylphenyl)-2-((2-((*E*)-(hydroxyimino)methyl)phenoxy)methyl)acrylate as a colourless solid. Single crystals suitable for X-ray diffraction were obtained by slow evaporation of a solution of the title compound in acetone at room temperature.

### Refinement

All H atoms were fixed geometrically and allowed to ride on their parent C atoms, with C—H distances fixed in the range 0.93–0.97 Å with *U*_iso_(H) = 1.5*U*_eq_(C) for methyl H 1.2*U*_eq_(C) for other H atoms.

### Crystal data

**Table:**

C_20_H_21_NO_4_	*Z* = 2
*M**_r_* = 339.38	*F*(000) = 360
Triclinic, *P*1	*D*_x_ = 1.273 Mg m^−^^3^
Hall symbol: -P 1	Mo *K*α radiation, λ = 0.71073 Å
*a* = 9.0053 (2) Å	Cell parameters from 6955 reflections
*b* = 9.3655 (3) Å	θ = 1.8–33.6°
*c* = 12.1793 (3) Å	µ = 0.09 mm^−^^1^
α = 75.299 (1)°	*T* = 293 K
β = 74.756 (1)°	Block, white
γ = 64.891 (1)°	0.25 × 0.22 × 0.19 mm
*V* = 885.43 (4) Å^3^	

### Data collection

**Table:**

Bruker APEXII CCD area detector diffractometer	6955 independent reflections
Radiation source: fine-focus sealed tube	4453 reflections with *I* > 2σ(*I*)
graphite	*R*_int_ = 0.023
ω and φ scans	θ_max_ = 33.6°, θ_min_ = 2.4°
Absorption correction: multi-scan (*SADABS*; Sheldrick, 1996)	*h* = −13→13
*T*_min_ = 0.978, *T*_max_ = 0.983	*k* = −14→14
24628 measured reflections	*l* = −18→18

### Refinement

**Table:**

Refinement on *F*^2^	Primary atom site location: structure-invariant direct methods
Least-squares matrix: full	Secondary atom site location: difference Fourier map
*R*[*F*^2^ > 2σ(*F*^2^)] = 0.053	Hydrogen site location: inferred from neighbouring sites
*wR*(*F*^2^) = 0.189	H-atom parameters constrained
*S* = 1.02	*w* = 1/[σ^2^(*F*_o_^2^) + (0.1052*P*)^2^ + 0.0753*P*] where *P* = (*F*_o_^2^ + 2*F*_c_^2^)/3
6955 reflections	(Δ/σ)_max_ < 0.001
229 parameters	Δρ_max_ = 0.31 e Å^−^^3^
0 restraints	Δρ_min_ = −0.29 e Å^−^^3^

### Special details

**Table:**

Geometry. All e.s.d.'s (except the e.s.d. in the dihedral angle between two l.s. planes) are estimated using the full covariance matrix. The cell e.s.d.'s are taken into account individually in the estimation of e.s.d.'s in distances, angles and torsion angles; correlations between e.s.d.'s in cell parameters are only used when they are defined by crystal symmetry. An approximate (isotropic) treatment of cell e.s.d.'s is used for estimating e.s.d.'s involving l.s. planes.
Refinement. Refinement of *F*^2^ against ALL reflections. The weighted *R*-factor *wR* and goodness of fit *S* are based on *F*^2^, conventional *R*-factors *R* are based on *F*, with *F* set to zero for negative *F*^2^. The threshold expression of *F*^2^ > σ(*F*^2^) is used only for calculating *R*-factors(gt) *etc*. and is not relevant to the choice of reflections for refinement. *R*-factors based on *F*^2^ are statistically about twice as large as those based on *F*, and *R*- factors based on ALL data will be even larger.

### Fractional atomic coordinates and isotropic or equivalent isotropic displacement parameters (Å^2^)

**Table:**

	*x*	*y*	*z*	*U*_iso_*/*U*_eq_	
C1	−0.16884 (13)	0.61894 (13)	0.41644 (9)	0.0372 (2)	
C2	−0.25120 (16)	0.53924 (17)	0.50619 (11)	0.0506 (3)	
H2	−0.3220	0.5916	0.5669	0.061*	
C3	−0.23045 (18)	0.38465 (18)	0.50735 (13)	0.0587 (4)	
H3	−0.2877	0.3337	0.5677	0.070*	
C4	−0.12419 (17)	0.30617 (16)	0.41841 (13)	0.0533 (3)	
H4	−0.1100	0.2017	0.4190	0.064*	
C5	−0.03793 (15)	0.38050 (15)	0.32794 (11)	0.0444 (3)	
H5	0.0334	0.3263	0.2682	0.053*	
C6	−0.05852 (12)	0.53598 (13)	0.32705 (9)	0.0354 (2)	
C7	−0.19037 (14)	0.78288 (14)	0.41442 (10)	0.0411 (2)	
H7	−0.1036	0.8170	0.3784	0.049*	
C8	0.13999 (13)	0.54048 (14)	0.15190 (10)	0.0404 (2)	
H8A	0.0841	0.5207	0.1027	0.048*	
H8B	0.2179	0.4387	0.1839	0.048*	
C9	0.23062 (13)	0.64543 (14)	0.08369 (9)	0.0376 (2)	
C10	0.39509 (14)	0.61395 (15)	0.11024 (10)	0.0420 (3)	
C11	0.58541 (18)	0.4676 (2)	0.23780 (14)	0.0680 (4)	
H11A	0.6696	0.4611	0.1697	0.102*	
H11B	0.6146	0.3663	0.2880	0.102*	
H11C	0.5773	0.5483	0.2769	0.102*	
C12	0.17940 (13)	0.76549 (14)	−0.00239 (10)	0.0400 (2)	
H12	0.2523	0.8176	−0.0358	0.048*	
C13	0.02946 (14)	0.82890 (14)	−0.05290 (10)	0.0389 (2)	
C14	−0.11884 (15)	0.80690 (17)	−0.00057 (11)	0.0474 (3)	
H14	−0.1272	0.7486	0.0734	0.057*	
C15	−0.25321 (16)	0.87026 (18)	−0.05692 (12)	0.0516 (3)	
H15	−0.3495	0.8518	−0.0205	0.062*	
C16	−0.24822 (16)	0.96077 (15)	−0.16646 (11)	0.0459 (3)	
C17	−0.10288 (17)	0.98621 (16)	−0.21751 (11)	0.0492 (3)	
H17	−0.0966	1.0479	−0.2903	0.059*	
C18	0.03252 (15)	0.92197 (15)	−0.16260 (11)	0.0468 (3)	
H18	0.1284	0.9410	−0.1994	0.056*	
C19	−0.3973 (2)	1.0316 (2)	−0.22612 (15)	0.0637 (4)	
H19A	−0.4829	1.1184	−0.1881	0.076*	
H19B	−0.3646	1.0766	−0.3051	0.076*	
C20	−0.4694 (3)	0.9162 (3)	−0.2271 (2)	0.0898 (7)	
H20A	−0.3902	0.8366	−0.2726	0.135*	
H20B	−0.5691	0.9716	−0.2597	0.135*	
H20C	−0.4954	0.8656	−0.1496	0.135*	
N1	−0.32728 (13)	0.87907 (12)	0.46171 (9)	0.0449 (2)	
O1	−0.32289 (13)	1.02929 (12)	0.45450 (10)	0.0588 (3)	
H1	−0.4168	1.0924	0.4764	0.088*	
O2	0.01993 (10)	0.62061 (9)	0.24319 (7)	0.0415 (2)	
O3	0.42776 (12)	0.50810 (14)	0.20593 (8)	0.0585 (3)	
O4	0.49093 (12)	0.67314 (14)	0.05241 (10)	0.0682 (3)	

### Atomic displacement parameters (Å^2^)

**Table:**

	*U*^11^	*U*^22^	*U*^33^	*U*^12^	*U*^13^	*U*^23^
C1	0.0339 (4)	0.0404 (5)	0.0323 (5)	−0.0132 (4)	−0.0039 (4)	−0.0013 (4)
C2	0.0490 (6)	0.0528 (7)	0.0386 (6)	−0.0196 (5)	0.0047 (5)	−0.0010 (5)
C3	0.0605 (8)	0.0540 (8)	0.0535 (8)	−0.0303 (6)	0.0014 (6)	0.0081 (6)
C4	0.0555 (7)	0.0415 (6)	0.0603 (8)	−0.0233 (5)	−0.0075 (6)	0.0018 (6)
C5	0.0447 (6)	0.0403 (6)	0.0466 (6)	−0.0179 (5)	−0.0036 (5)	−0.0063 (5)
C6	0.0327 (4)	0.0379 (5)	0.0325 (5)	−0.0136 (4)	−0.0053 (4)	−0.0010 (4)
C7	0.0406 (5)	0.0453 (6)	0.0345 (5)	−0.0171 (4)	−0.0008 (4)	−0.0064 (4)
C8	0.0390 (5)	0.0399 (6)	0.0396 (6)	−0.0159 (4)	0.0031 (4)	−0.0110 (4)
C9	0.0359 (5)	0.0424 (6)	0.0343 (5)	−0.0167 (4)	0.0024 (4)	−0.0119 (4)
C10	0.0379 (5)	0.0475 (6)	0.0390 (6)	−0.0158 (5)	−0.0017 (4)	−0.0107 (5)
C11	0.0469 (7)	0.0909 (12)	0.0560 (9)	−0.0153 (7)	−0.0158 (6)	−0.0077 (8)
C12	0.0379 (5)	0.0447 (6)	0.0386 (6)	−0.0193 (4)	−0.0002 (4)	−0.0090 (4)
C13	0.0404 (5)	0.0404 (6)	0.0371 (5)	−0.0178 (4)	−0.0027 (4)	−0.0085 (4)
C14	0.0444 (6)	0.0608 (7)	0.0360 (6)	−0.0254 (5)	−0.0049 (4)	0.0009 (5)
C15	0.0445 (6)	0.0660 (8)	0.0458 (7)	−0.0289 (6)	−0.0081 (5)	0.0021 (6)
C16	0.0494 (6)	0.0448 (6)	0.0456 (6)	−0.0199 (5)	−0.0126 (5)	−0.0032 (5)
C17	0.0578 (7)	0.0466 (7)	0.0417 (6)	−0.0251 (6)	−0.0094 (5)	0.0052 (5)
C18	0.0466 (6)	0.0471 (7)	0.0460 (6)	−0.0244 (5)	−0.0041 (5)	0.0008 (5)
C19	0.0647 (9)	0.0652 (9)	0.0617 (9)	−0.0253 (7)	−0.0287 (7)	0.0077 (7)
C20	0.0947 (13)	0.1082 (15)	0.0893 (13)	−0.0635 (12)	−0.0580 (11)	0.0331 (11)
N1	0.0464 (5)	0.0425 (5)	0.0423 (5)	−0.0165 (4)	0.0006 (4)	−0.0104 (4)
O1	0.0607 (6)	0.0479 (5)	0.0677 (7)	−0.0234 (4)	0.0043 (5)	−0.0207 (5)
O2	0.0445 (4)	0.0389 (4)	0.0361 (4)	−0.0188 (3)	0.0074 (3)	−0.0080 (3)
O3	0.0459 (5)	0.0795 (7)	0.0437 (5)	−0.0238 (5)	−0.0092 (4)	0.0013 (5)
O4	0.0510 (5)	0.0811 (7)	0.0752 (7)	−0.0386 (5)	−0.0171 (5)	0.0124 (6)

### Geometric parameters (Å, °)

**Table:**

C1—C2	1.3898 (15)	C11—H11B	0.9600
C1—C6	1.4076 (15)	C11—H11C	0.9600
C1—C7	1.4582 (17)	C12—C13	1.4579 (16)
C2—C3	1.376 (2)	C12—H12	0.9300
C2—H2	0.9300	C13—C14	1.3969 (15)
C3—C4	1.375 (2)	C13—C18	1.3984 (16)
C3—H3	0.9300	C14—C15	1.3812 (18)
C4—C5	1.3862 (17)	C14—H14	0.9300
C4—H4	0.9300	C15—C16	1.3883 (18)
C5—C6	1.3851 (16)	C15—H15	0.9300
C5—H5	0.9300	C16—C17	1.3848 (18)
C6—O2	1.3621 (12)	C16—C19	1.5112 (19)
C7—N1	1.2692 (15)	C17—C18	1.3777 (19)
C7—H7	0.9300	C17—H17	0.9300
C8—O2	1.4392 (13)	C18—H18	0.9300
C8—C9	1.4940 (15)	C19—C20	1.482 (2)
C8—H8A	0.9700	C19—H19A	0.9700
C8—H8B	0.9700	C19—H19B	0.9700
C9—C12	1.3387 (17)	C20—H20A	0.9600
C9—C10	1.4893 (16)	C20—H20B	0.9600
C10—O4	1.1985 (14)	C20—H20C	0.9600
C10—O3	1.3320 (16)	N1—O1	1.4039 (14)
C11—O3	1.4380 (17)	O1—H1	0.8200
C11—H11A	0.9600		
			
C2—C1—C6	118.34 (11)	H11B—C11—H11C	109.5
C2—C1—C7	121.91 (10)	C9—C12—C13	131.31 (10)
C6—C1—C7	119.73 (9)	C9—C12—H12	114.3
C3—C2—C1	121.56 (12)	C13—C12—H12	114.3
C3—C2—H2	119.2	C14—C13—C18	116.77 (11)
C1—C2—H2	119.2	C14—C13—C12	125.70 (11)
C4—C3—C2	119.34 (12)	C18—C13—C12	117.53 (10)
C4—C3—H3	120.3	C15—C14—C13	121.07 (11)
C2—C3—H3	120.3	C15—C14—H14	119.5
C3—C4—C5	120.98 (12)	C13—C14—H14	119.5
C3—C4—H4	119.5	C14—C15—C16	121.71 (11)
C5—C4—H4	119.5	C14—C15—H15	119.1
C6—C5—C4	119.63 (12)	C16—C15—H15	119.1
C6—C5—H5	120.2	C17—C16—C15	117.40 (12)
C4—C5—H5	120.2	C17—C16—C19	121.52 (12)
O2—C6—C5	124.79 (10)	C15—C16—C19	121.07 (12)
O2—C6—C1	115.07 (10)	C18—C17—C16	121.33 (12)
C5—C6—C1	120.13 (10)	C18—C17—H17	119.3
N1—C7—C1	120.10 (10)	C16—C17—H17	119.3
N1—C7—H7	120.0	C17—C18—C13	121.68 (11)
C1—C7—H7	120.0	C17—C18—H18	119.2
O2—C8—C9	108.04 (9)	C13—C18—H18	119.2
O2—C8—H8A	110.1	C20—C19—C16	114.28 (13)
C9—C8—H8A	110.1	C20—C19—H19A	108.7
O2—C8—H8B	110.1	C16—C19—H19A	108.7
C9—C8—H8B	110.1	C20—C19—H19B	108.7
H8A—C8—H8B	108.4	C16—C19—H19B	108.7
C12—C9—C10	115.74 (10)	H19A—C19—H19B	107.6
C12—C9—C8	125.94 (11)	C19—C20—H20A	109.5
C10—C9—C8	118.30 (10)	C19—C20—H20B	109.5
O4—C10—O3	122.56 (11)	H20A—C20—H20B	109.5
O4—C10—C9	125.05 (12)	C19—C20—H20C	109.5
O3—C10—C9	112.38 (10)	H20A—C20—H20C	109.5
O3—C11—H11A	109.5	H20B—C20—H20C	109.5
O3—C11—H11B	109.5	C7—N1—O1	111.99 (10)
H11A—C11—H11B	109.5	N1—O1—H1	109.5
O3—C11—H11C	109.5	C6—O2—C8	117.90 (9)
H11A—C11—H11C	109.5	C10—O3—C11	116.58 (11)
			
C6—C1—C2—C3	−1.8 (2)	C9—C12—C13—C14	19.8 (2)
C7—C1—C2—C3	179.87 (13)	C9—C12—C13—C18	−161.25 (12)
C1—C2—C3—C4	0.8 (2)	C18—C13—C14—C15	2.05 (19)
C2—C3—C4—C5	0.0 (2)	C12—C13—C14—C15	−178.95 (13)
C3—C4—C5—C6	0.2 (2)	C13—C14—C15—C16	−1.3 (2)
C4—C5—C6—O2	179.74 (12)	C14—C15—C16—C17	−0.3 (2)
C4—C5—C6—C1	−1.19 (18)	C14—C15—C16—C19	−178.94 (14)
C2—C1—C6—O2	−178.86 (11)	C15—C16—C17—C18	1.1 (2)
C7—C1—C6—O2	−0.51 (15)	C19—C16—C17—C18	179.70 (13)
C2—C1—C6—C5	1.98 (17)	C16—C17—C18—C13	−0.3 (2)
C7—C1—C6—C5	−179.66 (11)	C14—C13—C18—C17	−1.29 (19)
C2—C1—C7—N1	−30.13 (18)	C12—C13—C18—C17	179.63 (12)
C6—C1—C7—N1	151.58 (11)	C17—C16—C19—C20	131.30 (18)
O2—C8—C9—C12	−84.39 (14)	C15—C16—C19—C20	−50.1 (2)
O2—C8—C9—C10	97.11 (11)	C1—C7—N1—O1	177.99 (10)
C12—C9—C10—O4	−9.75 (18)	C5—C6—O2—C8	−3.60 (16)
C8—C9—C10—O4	168.90 (12)	C1—C6—O2—C8	177.30 (9)
C12—C9—C10—O3	171.59 (10)	C9—C8—O2—C6	−169.80 (9)
C8—C9—C10—O3	−9.75 (15)	O4—C10—O3—C11	0.3 (2)
C10—C9—C12—C13	179.15 (11)	C9—C10—O3—C11	178.95 (12)
C8—C9—C12—C13	0.6 (2)		

### Hydrogen-bond geometry (Å, °)

**Table:**

Cg2 is the centroid of the C13–C18 ring.

**Table:**

*D*—H···*A*	*D*—H	H···*A*	*D*···*A*	*D*—H···*A*
O1—H1···N1^i^	0.82	2.15	2.8568 (15)	145.
C8—H8B···O3	0.97	2.34	2.7146 (18)	102
C12—H12···O4	0.93	2.37	2.7742 (18)	106
C14—H14···O2	0.93	2.51	3.3002 (16)	143
C15—H15···O4^ii^	0.93	2.50	3.3524 (16)	152.
C5—H5···Cg2^iii^	0.93	2.94	3.7756 (14)	150

Symmetry codes: (i) −*x*−1, −*y*+2, −*z*+1;  (ii) *x*−1, *y*, *z*; (iii) −*x*, −*y*+1, −*z*.
